# Relationship between H.Pylori infection and clinicopathological features and prognosis of gastric cancer

**DOI:** 10.1186/1471-2407-10-374

**Published:** 2010-07-17

**Authors:** Hai-Bo Qiu, Li-Yi Zhang, Rajiv-Prasad Keshari, Guo-Qiang Wang, Zhi-Wei Zhou, Da-Zhi Xu, Wei Wang, You-Qin Zhan, Wei Li

**Affiliations:** 1 State Key Laboratory of Oncology in South China, Guangzhou, Guangdong, 510060, PR China; 2 Department of Gastric and Pancreatic Surgery, Sun Yat-sen University Cancer Center, Guangzhou, Guangdong, 510060, PR China; 3 Department of Clinical Oncology, The University of HongKong, HongKong

## Abstract

**Background:**

Aimed to assess the relationship between H.Pylori and the clinicopathological features and prognosis of gastric cancer by quantitative detection of H.Pylori.

**Methods:**

157 patients were enrolled, all patients had a record of clinicopathological parameters. Specimens including the tumor and non-neoplastic were detected for H.Pylori by Real-Time PCR and analyzed clinical data retrospectively. Variables independently affecting prognosis were investigated by means of multivariate analysis using the Cox proportional hazards model.

**Results:**

H.Pylori infection was greater in non-neoplastic tissue than the tumor tissue (p < 0.05), H.Pylori infection and its copies were related to the tumor site and N staging (p < 0.05). Overall survival (OS) in all 157 patients has no correlation with the H.Pylori infection status (p = 0.715). As to the patients who underwent a curative surgery, relapse-free survival (RFS) has no correlation with the H.Pylori infection status (p = 0.639). Among the H.Pylori positive patients, OS and RFS of those with higher copies were longer than in patients with low copies, but there was no significant statistical difference.

**Conclusions:**

H.Pylori infection status and its copies were related to N staging. The OS and RFS in patients with positive H.Pylori status has no significant difference from the patients with negative H.Pylori status.

## Background

Gastric cancer is the second most common cause of cancer death, though the incidence has decreased dramatically in some developed countries over the past decades [1], patients with this disease commonly have a poor outlook[2]. Even after potentially curative surgery, more than half the patients have tumor recurrence. Lymph node involvement, depth of invasion, age, and tumor location have been identified as the most significant clinicopathologic prognostic factors[3,4]. However, NCCN Clinical Practice Guideline of Gastric Cancer suggests that patients with gastric cancer should eradicate Helicobacter Pylori (H.Pylori) to reduce the risk of recurrence, which was defined as class evidence[5].

H.Pylori, a Gram-negative microaerophilic spiral bacterium, was first proposed to be associated with gastric cancer by co-discoverer Marshall in 1983[6]. In 1994, the International Agency for Research on Cancer (IARC) classified H.Pylori as a definite classI carcinogen[7,8]. After that, a lot of studies have been carried out, including epidemiological investigations[9], experimental studies[10], animal experimental data[11,12], case control clinical trials[13,14]. Most of these studies strengthened the fact that H.Pylori is the main cause of gastric adenocarcinoma. However, this point of view is still controversial and more reliable data is required. How H.Pylori infection leads to gastric cancer is not yet completely clear, as a variety of pathogenic factors may act on different stages, such as virulence factors[15], DNA damage, host factors[16,17] and COX-2[18]. The incidence of gastric cancer is a multi-factor and multi-step process.

At present, there is no convincing data for the relationship between H.Pylori infection status or copies and the clinicopathological features. Besides, the prognosis differences in survival of patients who are positive for H.Pylori compared with those who are negative remains unknown. In a variable proportion of gastric cancer patients, H.Pylori infection cannot be detected and distinct clinical and pathologic features have been described in this subgroup [19,20]. Furthermore, a recent German study provided evidence of a better prognosis in patients with H.Pylori infection compared with negative cases[21] and an Italian study showed that negative H.Pylori status was associated with poor prognosis in patients with gastric cancer [22]. However, to our knowledge, these clinically relevant findings have been not yet been validated in large experiences from other centers. Most of these studies used serological analysis as the only method to assess H.Pylori status, a few of them even added the molecule method of PCR; so the results of these studies are hardly convincing. In fact, there are many types of methods provided for detecting the H.Pylori, including culture, histopathological diagnosis, urease test, molecule method, serological analysis and UBT, but only culture, Real-Time PCR and UBT have a high degree of sensitivity and specificity[23].

In the current study, we used the method of Real-Time PCR to detect H.Pylori infection status and its copies, and aimed to investigate the potential impact of H.Pylori status on the clinicopathological features of gastric cancer of patients who underwent surgery and were followed up for a significant period of time to assess differences in relapse-free survival and overall survival.

## Patients and Methods

### Patients

The study population consisted of 157 consecutive patients (107 men and 50 women) scheduled for surgery from 1 January 2002 to 31 December 2006 at Sun Yat-Sen University Cancer Center, GuangZhou, China. The median age of the patients was 57.2 y (range: 27-78 y), All patients enrolled in this study had a histologically confirmed diagnosis of primary gastric cancer that was confirmed pathologically after surgery. Patients who underwent non-resective surgery, and those with Siewert type I cardia adenocarcinoma were excluded from the study, Patients with H.pylori eradication therapy or treatment with antibiotics, bismuth-containing compounds, H2-receptor blockers or proton pump inhibitors within 4 weeks also had been excluded from the study. Surgery consisted of subtotal or total gastrectomy in all patients. A standardized technique was used for surgical resection and lymphadenectomy, as described elsewhere[3]. Age, sex, type of surgery, tumor location, TNM stage, chemotherapy, specimen length, tumor size, and tumor differentiation, were recorded for each patient in a database. Tumor length and the width of fresh specimens were recorded by the pathologists. pTNM classification followed the criteria of the 6th edition of the UICC[24]. All the patients who underwent any type of chemotherapy were on the 5-FU-based regimen, and the basic regimen was FOLFOX6 or XELOX. All Experimental research that is reported in the manuscript have obtained informed consent from the subjects of the study and been Ethically performed with the approval of an appropriate ethics committee in Sun Yat-sen University of Cancer Center.

Tumor tissue and non-neoplastic tissue from all patients was collected from the resected specimen in the operating room within 30 minutes after the removal of the stomach. Non-neoplastic tissue was removed from the normal gastric tissue at a distance of at least 5 cm from the tumor. When neoplasm involved the entire antrum, non-neoplastic mucosa was removed from the middle or upper third of the stomach. Specimens were immediately frozen in liquid nitrogen, processed, coded, and stored at -80°C in a tissue bank located in the laboratory of the scientific research department.

### DNA Extraction and Real-Time PCR for H.Pylori

Both tumor tissue and Non-neoplastic tissue obtained from all patients included in the study was retrieved from the tissue bank. DNA was extracted from 50 mg of solid tissue (both tumor and Non-neoplasitic) by using the QIAamp DNA stool minikit (Qiagen). In order to increase the yield of purified DNA, the whole supernatant (500 μl) was used for further DNA purification after the addition of the InhibitEX tablet, adapting the following steps of the Qiagen protocol to this altered sample amount. From H.Pylori isolates, DNA was extracted by using the QIAamp DNA minikit according to the protocol for isolation of genomic DNA from bacterial cultures (Qiagen).

GenBank was searched for sequences of the genes encoding 16S rRNA of H.Pylori. The published sequences were aligned by using CLUSTALW http://www.ebi.ac.uk/clustalw/, and primers and probes were designed by using Primer Express software (Perkin-Elmer/Applied Biosystems, Foster City, Calif.) and the LightCycler probe design software (Roche Diagnostics, Mannheim, Germany). A BLAST search was performed to check the specificity of the DNA sequences of the primers and probes http://www.ncbi.nlm.nih.gov/BLAST/.

The assay contained two PCR primers (DaAn Gene Co., Ltd. of Sun Yat-sen University, GuangZhou, China): primer1 F(5'- GCT AAG AGA TCA GCC TAT GTC -3') and primer2 R(5'- CCG TGT CTC AGT TCC AGT GT- 3') designed to amplify a 118 base pair fragment of 16S rDNA, together with a probe, Tensensor (5'-LCRed705-GCA TGT GGT TTA ATT CGA AGA TAC ACphos- 3'), labelled with the fluorophore LC Red 705 (DaAn Gene Co., Ltd. of Sun Yat-sen University, GuangZhou, China).

Each Real-Time PCR was performed in a final volume of 50 μL containing a buffer of 10 μL(10 mM Tris-HCl(PH8.0), 50 mM KCl and 1.5 mM MgCl_2 _), 2 μL of cDNA, 1 μl of each primer(F 10 pmol/μl, R 10 pmol/μl), 1 μl of each deoxynucleotide(10 mM), 1 μl of Taq polymerase (3U/μl) (DaAn Gene Co., Ltd. of Sun Yat-sen University, GuangZhou, China) and 1 μl probe(DaAn Gene Co., Ltd. of Sun Yat-sen University, GuangZhou, China). Each reaction mixture was amplified as follows: denaturation at 93°C for 3 minutes followed by 40 cycles of denaturation at 93°C for 30 seconds; annealing at 55°C for 45 seconds; and extension at 72°C for 40 seconds. Amplifications were performed with an ABI 7500 DNA engine (Applied Biosystems Inc, CA, USA). Used H.Pylori standard amplification curves to make the standard curve linear regression graph, and then changed the Ct values into the number of H.Pylori copies.

### Follow up

All patients, after discharge from the hospital, entered a follow-up program according to standard protocol[25]. Within the first 2 years after surgery, a follow-up every 3 months consisted of a clinical examination, routine blood tests, assessment of concentration of tumor markers, and abdominal ultrasonography or CT scan; endoscopy was done every 6 months for the first 2 years after surgery. For the next 3 years, patients were followed up every 6 months and underwent endoscopy every 12 months. At relapse (defined as local recurrence or metastasis at distant sites), all patients were staged fully to detect disease at other sites. Survival was measured from the date of tumor resection to death or to the last date the patient was known to be alive. The follow-up was closed in December 2008. The mid follow-up period was 24.4 months (range, 0.2-81.8 months).

### Statistical Analysis

Cases were considered positive for H.Pylori when the 16S rRNA was present on Real-Time PCR product amplification in either tumor tissue or the Non-neoplastic tissue, and the cases were defined as positive for H.Pylori in normal tissue when the 16S rRNA was present on Real-Time PCR product amplification in Non-neoplastic tissue. All patients were divided into two groups; Group A of 82 patients was comprised of patients who were positive for H.Pylori infection, Group B of 75 patients was comprised of patients who had negative results for Real-Time PCR product amplification. Furthermore, Group A was divided into two groups by the median(1338.5copies) copies number of H.Pylori, one group of HP infection with high-copies (≥1338.5 copy number) of 41 cases, the other group of HP infection with low-copies (<1338.5 copy number) of 41 cases.

Statistical analysis was performed by using SPSS software for Windows (version 13.0; SPSS Inc, Chicago, IL). The association between H-pylori infection status and clinicopathological features was compared using Pearson Chi-square test (2-tailed tests in each). The independent-sample Student t test was used for comparison of age at time of surgery. Relapse-free survival was calculated for all patients who had not had local or distant relapse and overall survival was computed for those patients who were alive, irrespective of relapse status. Long-term survival was evaluated using the Kaplan-Meier method, considering death or relapse from cancer as the endpoint; patients who died of causes other than tumor recurrence were considered as censored at the time of death. The log-rank test was used for statistical comparisons between factors. Variables independently affecting prognosis were investigated by means of multivariate analysis using the Cox proportional hazards model. The score statistic was used to select variables for entry into the model. A statistical level of P < 0.05 was used for the inclusion of prognostic variables.

## Results

### H.Pylori Status as Determined by Real-Time PCR

**Figure 1:**
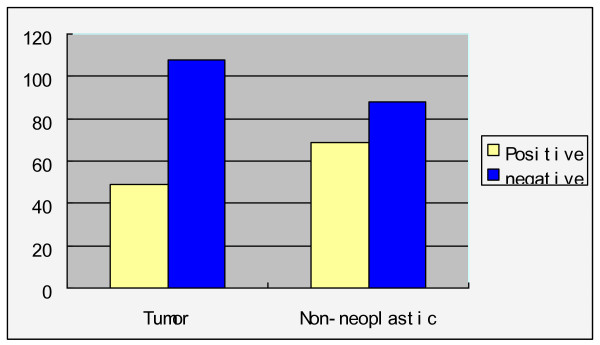
**Detection of H.Pylori in tumor and Non-neoplastic tissue (two groups comparison P = 0.003)**.

Quantitative detection of H.Pylori from 157 cases of patients with gastric cancer, each including tumor and non-neoplastic tissue; a total of 314 specimens. 49 patients (31.2%) demonstrated positive H.Pylori in the tumor tissue, and 69 patients (43.9%) demonstrated positive in the non-neoplastic tissue (Figure 1). The combined positive in both tumor and non-neoplastic tissue is 36 patients (22.9%), while 82(52.2%) patients can be detected with H.Pylori in any one of the specimens. The number of H.Pylori copies ranges from 2.51 × 10^2^~1.45 × 10^8^.

### The Association between Clinicopathologic Features and H.Pylori Infection Status

Table 1 shows the results for a comparison of the clinical and pathologic features between the patients in Group A and Group B. Of the patients in Group A, neoplasms were more frequently located in the upper stomach (37.8% vs 18%), and a more advanced pN classification(N2/N3) was observed while a more lower pN classification(N0/N1) was seen in group B. Before surgery, patients who were negative for H.Pylori had higher concentrations of carcinoembryonic antigen(CEA) than did those who were positive for H.Pylori. No statistical significant differences with regard to gender, age, tumor size, tumor site, gross type, depth of tumor invasion, distant metastasis, UICC stage, CA199 and the P53 gene expression were observed between the two groups.

**Table 1:** Association Between Clinicopathologic Features and H.Pylori Status

Clinicopathological Features	Group A (Positive) (n = 82)(%)	Group B (Negative) (n = 75)(%)	P value
Gender			0.287
Male	59(72.0)	48(64.0)	
Female	23(38.0)	27(36.0)	
Age			0.128
<57.2 years	45(54.9)	32(42.7)	
≥57.2 years	37(45.1)	43(57.8)	
Tumor site			0.040
Upper	31(37.8)	18(24.0)	
Middle or Lower	51(62.2)	57(76.0)	
Tumor size (Mean ± SD)	5.64 ± 2.82	5.84 ± 2.99	0.671
Gross type			0.816
Early GC I	1(1.2)	1(1.3)	
Early GC III	10(12.2)	7(9.3)	
Borrmann I	15(18.3)	12(16.0)	
Borrmann II	37(45.1)	32(42.7)	
Borrmann III	11(13.4)	15(20.0)	
Borrmann IV	8(9.8)	8(10.7)	
Histological Differentiate			0.236
Well	36(43.9)	28(37.3)	
Poorly	35(42.7)	31(41.3)	
Mucous/Signet-ring cell	11(13.4)	16(21.3)	
Depth of tumor invasion			0.749
T1	4(4.9)	2(2.7)	
T2	9(11.0)	9(12.0)	
T3	58(70.7)	54(72.0)	
T4	11(13.4)	10(13.3)	
Lymph-node metastasis			0.039
N0	30(36.6)	16(21.3)	
N1	29(35.4)	29(38.7)	
N2	14(17.1)	19(25.3)	
N3	9(11.0)	11(14.7)	
Distant metastases			0.822
M0	71(86.6)	64(84.0)	
M1	11(13.4)	11(16.0)	
UICC stage			0.124
I	10(12.2)	6(8.0)	
II	20(24.4)	13(17.3)	
III	32(39.0)	32(42.7)	
IV	20(24.4)	24(32.0)	
surgery			0.943
Curative	65(79.8)	62(85.3)	
palliative	17(20.7)	11(14.7)	
Tumor marker			
CEA			0.026
<5 μg/L	64(11.0)	51(68.0)	
≥5 μg/L	9 (78.0)	19(25.3)	
CA199			0.346
<30 μg/L	44(53.7)	44(58.7)	
≥30 μg/L	12(14.6)	18(24.0)	
P53			0.827
positive	9(11.0)	8(10.7)	
negative	40(48.8)	40(53.3)	

### The Association between Clinicopathologic Features and H.Pylori Copies

The three clinicopathological features mentioned above that were associated with the H.Pylori infection status were analyzed to see whether they were associated with the H.Pylori Copies. In Table 2, a comparison of the clinical and pathologic features between the patients in Group 1 with high-copies H.Pylori and Group 2 with low-copies H.Pylori is reported.

**Table 2:** The Association between Clinicopathologic Features and H.Pylori Copies

	H.Pylori high-titer (41cases,%)	H.Pylori low-titer (41cases,%)	P value
Tumor site			0.013
Upper	21(51.2)	10(24.4)	
Middle or Lower	20(48.8)	31(75.6)	
Lymph-node metastasis			0.014
N0	20(48.8)	10(24.4)	
N1	13(31.7)	16(39.0)	
N2	6(14.6)	8(19.5)	
N3	2(4.9)	7(17.1)	
CEA			0.269
<5 μg/L	34(82.9)	30(73.2)	
≥ 5 μg/L	3(7.3)	6(14.6)	

Of the patients in the group with high-copies H.Pylori, neoplasms were more frequently located in the upper stomach (51.2% vs 18%), and a more lower pN classification(N0/N1) was observed while a more advanced pN classification(N0/N1) was seen in the group with low-copies H.Pylori. However, no statistical significant difference was observed in the concentrations of CEA between the two groups (Table 2).

### H.Pylori infection and Survival

The potential impact of H.Pylori status on the long-term survival of patients was investigated by means of univariate and multivariate analysis. Overall Survival analysis was performed in 157 patients who underwent surgery. At the end of follow-up, 75 patients were still alive, 82 patients had died of tumor, and 1 patient had died of other causes; the cancer-related 5-year survival rate in the entire series was 48.4%.

**Figure 2:**
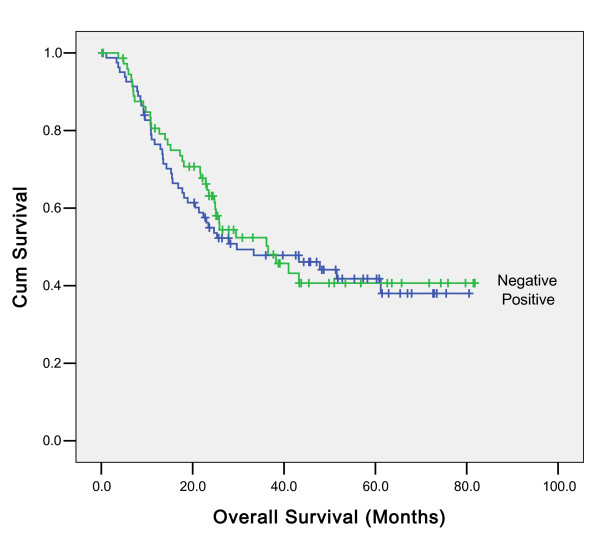
**Association between overall survival and H.Pylori infection Status of 157 patients with gastric cancer (p = 0.715)**.

**Figure 3:**
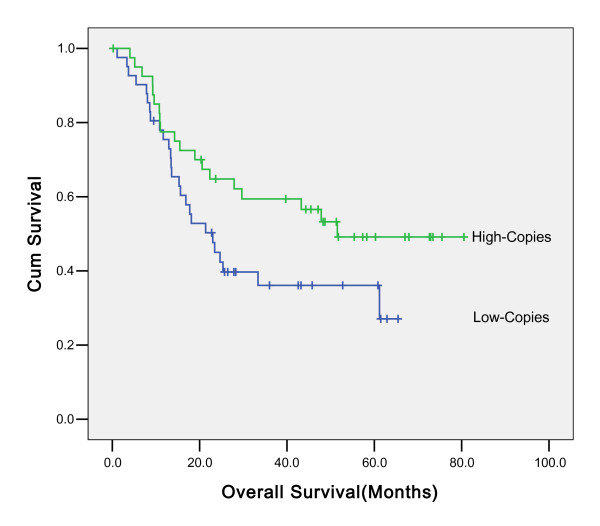
**Association between overall survival and the number of H.Pylori copies in patients who were positive for H.Pylori (P = 0. 068)**.

Univariate analyses showed an association between overall survival and histological differentiate, surgery, UICC stage and concentration of CA-199(data not shown). However, such association cannot be found between H.Pylori infection status and overall survival (p = 0.715) (Figure 2). In multivariate analyses, histological differentiate, surgery and UICC stage were independent prognostic factors for overall survival. Concentration of CA-199 was not significantly associated with overall survival in multivariate analyses (data not shown). The effect of H.Pylori status on prognosis was observed in all the subgroups according to the UICC stage examined; the difference was not found to be statistically significant (data not shown), In addition, the effect of H.Pylori status on prognosis were evaluated in all the subgroups regard to the different tumor sites, the difference was not found to be statistically significant (Additional files 1 and 2). Further analysis for the patients who were positive for H.Pylori were performed; Kaplan Meier survival analysis showed that the overall survival in patients with high copies H.Pylori infection was better than in the patients with low copies, but the difference was not statistically significant (P = 0. 068) (Figure 3.) The association between H.Pylori copies and prognosis were evaluated in all the subgroups regard to the different tumor sites, There is no statistically significance (Additional files 3 and 4).

Relapse-Free Survival analysis was performed in 129 patients who underwent R0 resection. At the end of follow-up, 73 patients were still alive, 56 patients had died of tumor recurrence or distant metastasis. The potential impact of H.Pylori status on the relapse-free survival of patients was investigated by means of univariate and multivariate analysis.

**Figure 4:**
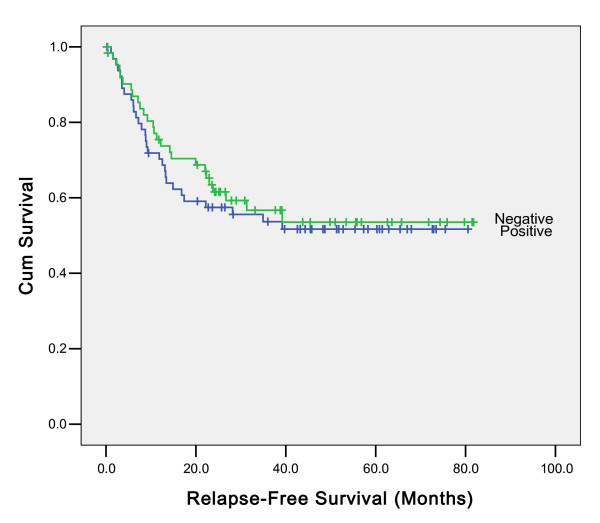
**Association between relapse-free survival and H.Pylori infection of 129 patients who underwent curative surgery (p = 0.639)**.

Univariate analyses showed an association between relapse-free survival and histological differentiate, tumor site, depth of tumor invasion (pT stage), Lymph-node metastasis (pN stage). However, such association cannot be found between H.Pylori infection status (p = 0.639) (Figure 4), the number of H.Pylori copies, H.Pylori infection in the non-neoplastic tissue and relapse-free survival. In multivariate analyses, histological differentiate, tumor site, depth of tumor invasion (pT stage), Lymph-node metastasis (pN stage) were independent prognostic factors for relapse-free survival. The effect of H.Pylori status on prognosis was observed in all the subgroups according to the UICC stage examined, the difference was not found to be statistically significant (data not shown). Data for overall survival of the patients who underwent R0 resection were almost the same as those for relapse-free survival. Histological differentiate, tumor site, depth of tumor invasion (pT stage), Lymph-node metastasis (pN stage) were independent prognostic factors for overall survival (data not shown).

## Discussion

Among the numerous methods described above, the most promising for the future is the application of Real-Time PCR [26]. The new real-time PCR technique is a breakthrough in the diagnosis of H.Pylori because it allows not only a quick and precise detection of H.Pylori but also its quantification [23]. In the method of Real-Time PCR, there are many genes to select for amplification, and the primer pair 16S rRNA and 23S rRNA showed a high specificity and sensitivity [27]. In the current study, we chose the 16S rRNA for amplification of Real-Time PCR; the results showed that 52.2% of 157 gastric cancer were positive for H.Pylori infection, which approached the results of other studies using the same method[28].

The main findings of the current study were: 1) H.Pylori infection was greater in non-neoplastic tissue than in tumor tissue; 2) H.Pylori infection was related to the tumor site, N staging, as well as the level of CEA; moreover, tumor site and N staging was related to the H.Pylori copies number. In Highly-infected patients, the N staging had an earlier stage(N0/N1) and tumor sites in upper stomach were found, while the CEA level was unrelated to the H.Pylori copies number; and 3) the overall survival and relapse-free survival in patients with positive H.Pylori status had no significant difference from the patients with negative H.Pylori status; and in H.Pylori positive patients, prognosis was better in those with high copies number of H.Pylori than in those with lower copies number, but the result was no significant statistical difference.

We recorded a higher frequency of infection with H.Pylori in non-neoplastic tissue than in the tumor tissue of the patients with gastric cancer. The possible explanation could be that 1) once a tumor arose in the location where the H.Pylori lived, the tumor would change the microenvironment, which would not be suitable for the H.Pylori to survive in any more.2) Some scholars [29]pointed out that the H.Pylori would be engulfed by the neutrophilic granulocyte, which is abundant in the neoplastic tissue. The host immune response to the tumor increases the induction of apoptosis by the bacteria.

The clinicopathological features of H.Pylori-positive patients were compared with those of the group of patients with negative H.Pylori status. Statistical analysis revealed that upper stomach location was more frequent in H.Pylori-positive patients; among the patients who were positive for H.Pylori, the copies of H.Pylori in upper stomach location were also higher than in the middle or lower stomach. In general, the stomach, especially the gastric pylorus is the most suitable place for H.Pylori to grow. Diagnosis and quantity of H.pylori infection was performed using non-neoplastic tissue at a distance of at least 5 cm from tumor. Therefore, sampling sites were different according to tumor site, for example, the upper stomach cancer may sample non-neoplastic tissue from the middle or lower stomach, and the middle or lower stomach cancer may sample from the upper. Furthermore, One point sampling from antrum or corpus may result in sampling error of H.pylori diagnosis and assessment of H.pylori amount, so we think this result is not able to conclude that H.Pylori infection was related to the tumor site.

We also found a higher rate of more advanced pN stage in H.Pylori-negative patients; furthermore, pN staging was related to the H.Pylori copies number. In the high-copies patients, the N staging has an earlier stage (N0/N1). Theoretically, tumor invasion and metastasis associated with the matrix metalloproteinases (MMPs) secreted by the gastric cancer cell, which can degrade extracellular matrix, change the structure of vascular basement membrane and promote cancer cell invasion and metastasis[30,31]. MMP-2 is one of the most important MMPs in the process of tumor invasion and metastasis. Monig et al.[32] reported that MMP-2 expression has a strong correlation with progress of lymph node metastasis of gastric cancer. Once H.Pylori has infected the stomach, it can stimulate the secretion of MMP-1, MMP-2, MMP-3 and TIMP-3 from gastric cancer cell[33], and finally prompt and speed up the progress invasion and metastasis of gastric cancer. However, the current study showed a converse conclusion, so we suggest that the H.Pylori infection is a beneficial factor for lymph-node metastasis.

Data from the current study indicated that H.Pylori infection was related to the level of CEA in the host serum. To our knowledge, it is the first time to report such an influence of this factor on the level of CEA. Patients with positive H.Pylori status had a low level compared with those who were negative, but the level of CEA was unrelated to the H.Pylori copies number; a possible explanation was autoimmune responses induced by H.Pylori inhibited the growth of cancer cell[34], and finally decreased the level of CEA in the host serum.

Dating back to 1995, studies from Taiwan[35,36], to our knowledge, was the first to report the relationship between H.Pylori infection status and the outcome of the patients with gastric cancer, multivariate analysis demonstrated that only TNM stage was the independent prognostic factor; however, the definition of H.Pylori status was performed only by serology, the number of patients was small, and follow-up was rather short. In 2006, a prospective study identified H.Pylori status as an independent prognostic factor [21]. Culture examination, histologic analysis, and serologic assay were performed to define H.Pylori status. The better prognosis noted in patients with H.Pylori infection was explained on the basis of an improved immune response against the tumor [21]. It also has been assumed that because H.Pylori components mimic specific receptors or surface molecules on gastric epithelial cells, auto-antibodies could induce a cross-reaction against gastric cancer cells[34]. However, several authors have raised doubts regarding the real prognostic value of H.Pylori status, suggesting that H.Pylori negativity may be simply related to more advanced tumor progression [37,38]. A recent study in Italy[22] revealed that patients with negative H.Pylori status appeared to have a poor prognosis, but the methods of this study is also doubtful, since they used the combination methods PCR for vacA and Serologic analysis to define the status of H.Pylori infection. Theoretically, PCR can detect only one copy of the target DNA when tested in water, but most studies have shown that standard PCR sensitivity and efficacy were not so exact [23,39], and the serologic analysis cannot distinguish whether the patient is having a current infection or an infection of the past[23,40]; both of the methods will contribute in part to a false negative.

We have shown that the UICC stage, histological differentiate and surgery were the independent prognostic factors of the overall survival of all the patients with gastric cancer, and the tumor site, histological differentiate, depth of tumor invasion, lymph-node metastasis were the independent prognostic factors of the relapse-free survival for the patients who underwent curative surgery. However, the H.Pylori infection status was not an independent prognostic factor as there was no difference in the survival of these patients with or without H.Pylori, but further analysis of H.Pylori positive patients revealed that the prognosis of those with high copies number of H.Pylori was better than those with low copies number, but the result was no significant statistical difference.

In vaccination experiments[41] with cholera toxoid in human beings, H.Pylori infection acts as an adjuvant for the induction of a local B-cell response in gastric mucosa. Furthermore, presentation of tumor antigens in the setting of inflammation might induce stronger immune responses in the presence of H.Pylori, and the cellular immune response caused by H.Pylori displays a type-1 T-helper-cell (Th1) type[42]. Thus, if the relation between a type 1 response and antitumor activity is confirmed by further studies, H.Pylori might contribute to an improved immune response against the tumor. However, some scholars cast doubt on this hypothesis; whether the patients who were negative for H.Pylori infection obtain a better outcome or not demands more evidence [37]. In fact, the results of the current study do not confirm this hypothesis; we suggest that the H.Pylori infection in patients with gastric cancer can induce the host's immune responses to the tumor, and finally have an effect on the lymph-node metastasis, but the immune responses were not so strong as to show an advantage in the survival eventually.

A trend toward a higher survival rate was observed in patients with high-copies H.Pylori infection compared with patients with low-copies infection, but the difference was not found to be statistically significant. This trend may be because of a higher protective effect of actual infection on long-term survival. Potential associations between the copies of H.Pylori infection and long-term survival should be investigated in further studies to clarify whether high-copies H.Pylori infection could be associated with a better prognosis in patients with gastric cancer.

Another question of whether patients with H.Pylori infection should undergo eradication after surgery remains unresolved. A study from Uemura et al.[43] conducted a nonrandomized H.Pylori eradication trial in patients whose gastric cancer was removed by endoscopic resection after the long-term clinical and endoscopic follow-up. The final result found that H.Pylori eradication may improve neutrophil infiltration and intestinal metaplasia in the gastric mucosa and inhibit the development of new carcinomas. This study was based on patients with early gastric carcinoma, but whether these results were suited for advanced gastric cancer remains unclear. However, the results of the current study show that there is no difference of relapse-free survival in patients with or without H.Pylori infection in the non-neoplastic tissue.

## Conclusions

H.Pylori continues to be one of the most common bacterial infections in human. Although enormous progress has been made in studying the virulence factors of H.Pylori and their variation, this information has not yet been used in clinical practice. Associations between bacterial characteristics and disease risks have not yet been defined sufficiently well to guide the clinician in treatment decisions.

## Competing interests

The authors declare that they have no competing interests.

## Authors' contributions

The work presented here was carried out in collaboration between all authors. HQ and ZWZ defined the research theme. HBQ and LYZ designed methods and experiments, carried out the laboratory experiments and wrote the paper, RPK revised the manuscript, GQW and WW analyzed the data and interpreted the results. DZX, YQZ and WL co-worked on associated data collection, interpretation and discussed analyses. All authors have contributed to, seen and approved the manuscript.

## Pre-publication history

The pre-publication history for this paper can be accessed here:

http://www.biomedcentral.com/1471-2407/10/374/prepub

## Supplementary Material

Additional file 1 **Association between overall survival and H.Pylori infection Status of patients with antral cancer**. A figure to show association between overall survival and H.Pylori infection in antral cancer. Click here for file

Additional file 2 **Association between overall survival and H.Pylori infection Status of patients with corporal cancer**. A figure to show association between overall survival and H.Pylori infection in corporal cancer. Click here for file

Additional file 3 **Association between overall survival and H.Pylori infection Copies of patients with antral cancer**. A figure to show association between overall survival and H.Pylori infection Copies in antral cancer. Click here for file

Additional file 4 **Association between overall survival and H.Pylori infection Copies of patients with corporal cancer**. A figure to show a ssociation between overall survival and H.Pylori infection Copies in corporal cancer. Click here for file
