# Supplementary material for: Relationship between H.Pylori infection and clinicopathological features and prognosis of gastric cancer
Source: BMC Cancer. 2010 Jul 17;10:374. doi: 10.1186/1471-2407-10-374 (PMC2914705; doi:10.1186/1471-2407-10-374)
Supplement: Additional file 2 — Association between overall survival and H.Pylori infection Status of patients with corporal cancer. A figure to show association between overall survival and H.Pylori infection in corporal cancer. [file 1471-2407-10-374-S2.DOC]

Additional Figure 2: Association between overall survival and H.Pylori infection Status of patients with corporal cancer (p=0. 899)
