# Supplementary material for: Relationship between H.Pylori infection and clinicopathological features and prognosis of gastric cancer
Source: BMC Cancer. 2010 Jul 17;10:374. doi: 10.1186/1471-2407-10-374 (PMC2914705; doi:10.1186/1471-2407-10-374)
Supplement: Additional file 3 — Association between overall survival and H.Pylori infection Copies of patients with antral cancer. A figure to show association between overall survival and H.Pylori infection Copies in antral cancer. [file 1471-2407-10-374-S3.DOC]

Additional Figure 3: Association between overall survival and H.Pylori infection Copies of patients with antral cancer (p=0.320)
